# Investigation of Serum Human Epididymitis Protein 4 Level in Rats with Experimental Acute Pancreatitis

**DOI:** 10.5152/tjg.2023.22489

**Published:** 2023-06-01

**Authors:** Ali Acar, Hamit Yaşar Ellidağ, Kadir Balaban, Serkan Öcal, Arif Aslaner, Tuğrul Çakır

**Affiliations:** 1 Department of General Surgery, Antalya Training and Research Hospital, University of Health Sciences, Antalya, Turkey; 2 Department of Biochemistry, Antalya Training and Research Hospital, University of Health Sciences, Antalya, Turkey; 3 Department of Pathology, Antalya Training and Research Hospital, University of Health Sciences, Antalya, Turkey; 4 Department of Gastroenterology, Antalya Training and Research Hospital, University of Health Sciences, Antalya, Turkey

**Keywords:** Pancreas, acute pancreatitis, cerulein, HE4

## Abstract

**Background:**

We aimed to show whether the serum level of Human Epididymitis Protein 4 increases in rats with an experimental acute pancreatitis model created by cerulein.

**Methods:**

This study included 24 male Sprague–Dawley rats which were randomly divided into 4 groups each containing 6 rats. Control: the group treated with saline, Group 1: pancreatitis group created with cerulein at a total dose of 80 µg/kg, Group 2: pancreatitis group created with cerulein at a total dose of 120 µg/kg, Group 3: pancreatitis group created with cerulein at a total dose of 160 µg/kg.

**Results:**

There were statistically significant differences between edema, acinar necrosis, fat necrosis, and perivascular inflammation scores among the study groups. While the degree of all histopathological findings is lowest in the control group, pancreatic parenchyma damage increases as the amount of injected cerulein increases. There was no statistically significant difference between alanine aminotransferase, aspartate aminotransferase, and Human Epididymis Protein 4 values between study groups. On the other hand, there was a statistically significant difference between amylase and lipase values. The lipase value of the control group was significantly lower than the lipase value of the second and third groups. The amylase value of the control group was significantly lower than all other groups. The highest Human Epididymis Protein 4 value was measured as 104 pmol/L in the first pancreatitis group, where the severity of pancreatitis was mild.

**Conclusions:**

In the present study, it was concluded that the Human Epididymis Protein 4 value increased in the case of mild pancreatitis, but there is no correlation between the severity of pancreatitis and the Human Epididymis Protein 4 value.

**Main Points**

- It is the first experimental study in the literature to have examined the serum Human Epididymis Protein 4 (HE4) level changes in rats in acute pancreatitis induced with cerulein in a dose-dependent manner.
- No correlation between the severity of pancreatitis and HE4 values was found.
- The levels of HE4 can be found as elevated in mild pancreatitis.

## INTRODUCTION

Acute pancreatitis is the case where enzymes in the pancreas are activated after an inflammatory process that starts with etiological factors such as gallstones and alcohol, causing acinar cell damage in the pancreas.^1,2^ The etiology, pathogenesis, and especially the treatment of acute pancreatitis are still controversial, despite the accumulation of knowledge from many experimental and clinical studies conducted over the past century.^3^

Although the disease is benign, it is necessary to be careful in terms of morbidities that may occur. Acute pancreatitis is a broad-spectrum disease ranging from mild self-limiting symptoms to fulminant processes with multiorgan failure and high morbidity.

Approximately 15%-25% of all patients with acute pancreatitis develop severe acute pancreatitis accompanied by local and systemic complications, multiorgan failure, and pancreatic necrosis. Mortality rates were determined as 3% in mildly edematous form and 15% in acute necrotizing form.^4^ Therefore, early recognition of necrosis is very important in determining the severity and prognosis of the disease. Various laboratory parameters are being investigated for early diagnosis.

Many pharmacological agents are used to induce experimental pancreatitis. Cerulein is an analog of cholecystokinin that causes bile reflux into the pancreatic duct and thus pancreatitis by causing loosening of the Oddi sphincter and contractions of the gallbladder.^5^ Cerulein has been used in experimental pancreatitis models with intraperitoneal, intravenous, and subcutaneous administration.^6^

Human Epididymis Protein 4 (HE4) belongs to the Whey Acidic Proteins (WAP) family. It is also called WFDC2 (WAP-4 disulfide core domain). It is a secretory protein localized on chromosome 20q12–13.1, a region that harbors a locus of 14 genes encoding protein domains that have homology with WAP.^7,8^

In recent studies, HE4 has been shown to be a potential biomarker for ovarian cancer, lung adenocarcinoma, endometrial cancer, mesothelioma, transitional cell cancers, and breast cancer.^9-15^ In addition, there are studies showing that the HE4 level is increased in gastric and pancreatic adenocarcinomas.^16^

Some WFDC proteins with antiproteinase activity have also been correlated with inflammatory processes.^17^ Patients with chronic inflammation and autoimmune responses are at increased risk of malignancy.^18^ Given the close association between HE4 and inflammation,^19-21^ we hypothesized that HE4 may be associated with inflammatory processes like pancreatitis. However, the role and related action mechanisms of HE4 in pancreatitis remain obscure. In this experimental study, we planned to evaluate the serum HE4 level and histopathological changes in rats with experimental acute pancreatitis induced with cerulein in a dose-dependent manner. To the best of our knowledge, this is the first study in the literature to have examined the serum HE4 level changes in rats in experimental acute pancreatitis induced with cerulein in a dose-dependent manner.

## MATERIALS AND METHODS

### Study Design

Approval for this study was granted by the Animal Research Ethics Committee of Antalya Akdeniz University dated February 12, 2019, and numbered B.30.2.AKD.0.05.07.00/28.

All procedures were applied at Akdeniz University Experimental Medicine and Animal Laboratory in accordance with the principles of the National Guidelines for Experimental Use of Laboratory Animals.

### Animals

In this study, 24 male Sprague–Dawley rats weighing between 300 and 350 g were used. During both preoperative and postoperative periods, the rats were housed in cages with constant environmental conditions (temperature: 23°C and humidity: 55.5%) and fed with standard laboratory feed and tap water. Access to food was stopped for 12 hours and to water for 2 hours prior to anesthesia.

The rats were randomized into 4 groups consisting of 6 rats each; Control: 1 mL of saline was given intraperitoneally 4 times at 1-hour intervals. Group 1: intraperitoneal 20 μg/kg cerulein was given 4 times at 1-hour intervals. Group 2: intraperitoneal 30 μg/kg cerulein was given 4 times at 1-hour intervals. Group 3: 40 μg/kg cerulein was given intraperitoneally 4 times at 1-hour intervals.

### Surgery and Experimental Protocol

Twelve hours after the last dose of saline and cerulein, all animals were anesthetized with an intraperitoneal injection of 50 mg/kg ketamine and 20 mg/kg xylasin. Surgical intervention was performed on all animals by the same surgeon. After the hairs were cleaned in the supine position, the abdominal skin was wiped with povidone-iodine, and skin antisepsis was applied. A median laparotomy was performed. The pancreatic tissue was removed using the appropriate method.

### Biochemical Analysis

Blood samples taken from rats after laparotomy were placed in biochemistry tubes. Plasma was separated after centrifugation. Aspartate transaminase (AST), alanine aminotransferase (ALT), amylase, lipase, and HE4 were studied from plasma.

Rat serum ALT, AST, lipase, and amylase levels were determined using an autoanalyzer (Beckman AU5800; Beckman Coulter Diagnostics, Brea, Calif, USA).

### Measurement of Human Epididymis Protein 4

Rat HE4 levels were quantified using a commercially available ELISA kit (MyBioSource, Inc. San Diego, Calif, USA) according to the manufacturer’s instructions. The sensitivity of the assay was 1 pmol/L with inter- and intra-assay coefficients of variation of <15%. The detection range of this kit is 6.25 pmol/L to 200 pmol/L. Assay results were expressed as pmol/L.

### Histopathological Evaluation

For examination, pancreatic tissues were fixed in 10% formaldehyde for microscopic monitoring and were routinely followed. Sections sent from tissues embedded in paraffin blocks were stained with hematoxylin–eosin (H–E) dye and evaluated under a light microscope. The evaluation was made by means of Schmidt pancreatic injury scoring.^17^ Edema, acinar necrosis, hemorrhage and fat necrosis, inflammation, and perivascular infiltration types were scored by giving values between 0 and 4 by making a single evaluation.

### Statistical Analysis

Data from Statistical Package for Social Sciences version 18.0 (SPSS Inc.; Chicago, IL, USA) were analyzed using the software of 1989 and 2010. The range of data is given with median, minimum and maximum values. The suitability of continuous variables to normal distributions was examined with the Shapiro–Wilk test, and it was found that all measurement values did not show a normal distribution. Since the parametric test assumptions were not provided, the Kruskal–Wallis test was used for comparisons of more than 2 groups. When a significant difference was determined, the Mann–Whitney *U* test with Bonferroni correction was used in post hoc paired comparisons for the source of the difference. The statistical significance level was accepted as P = .05 in the study.

## Results

**Figure 1.**
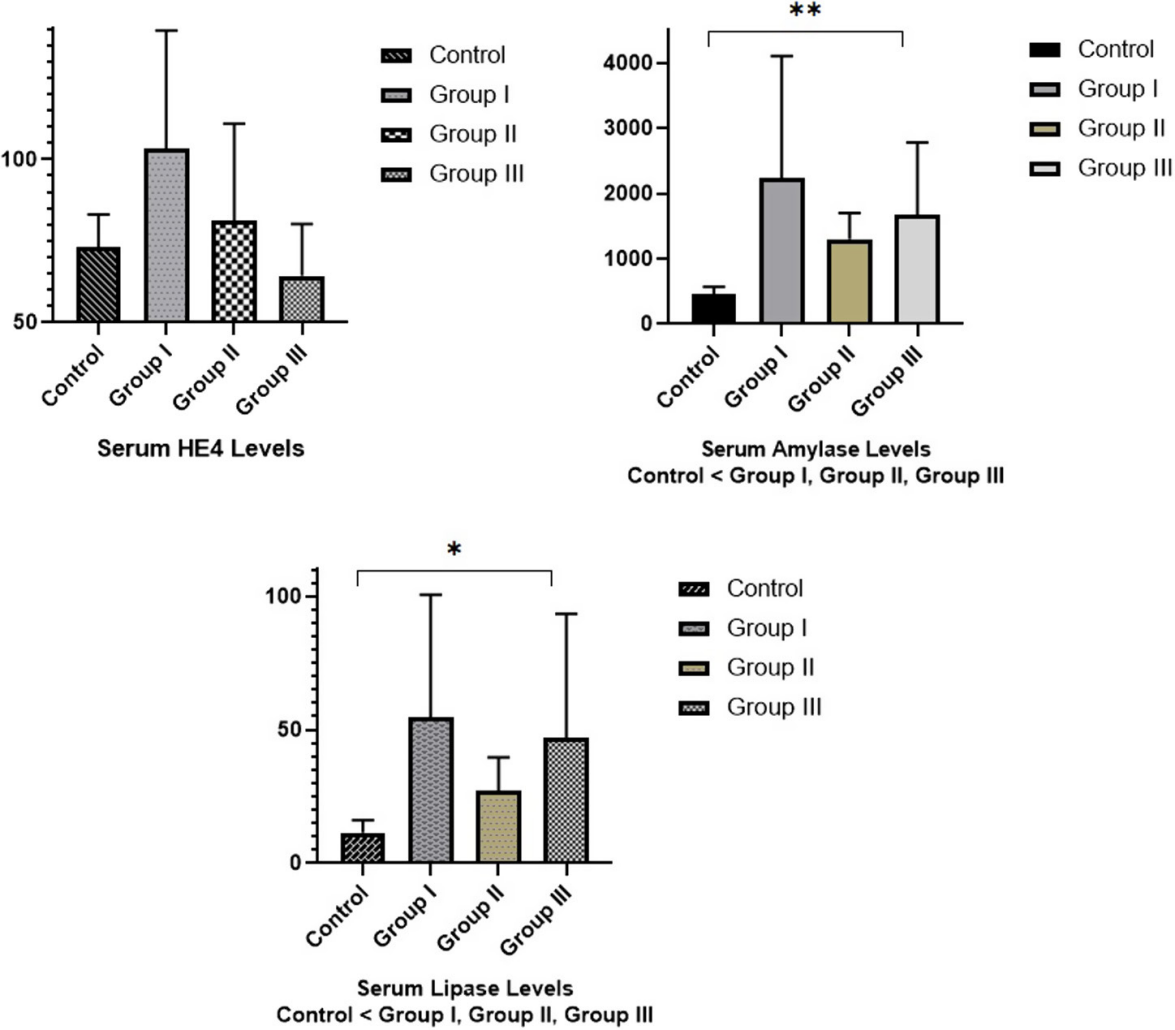
Distribution of HE4, serum amylase and lipase values among study groups.

**Figure 2.**
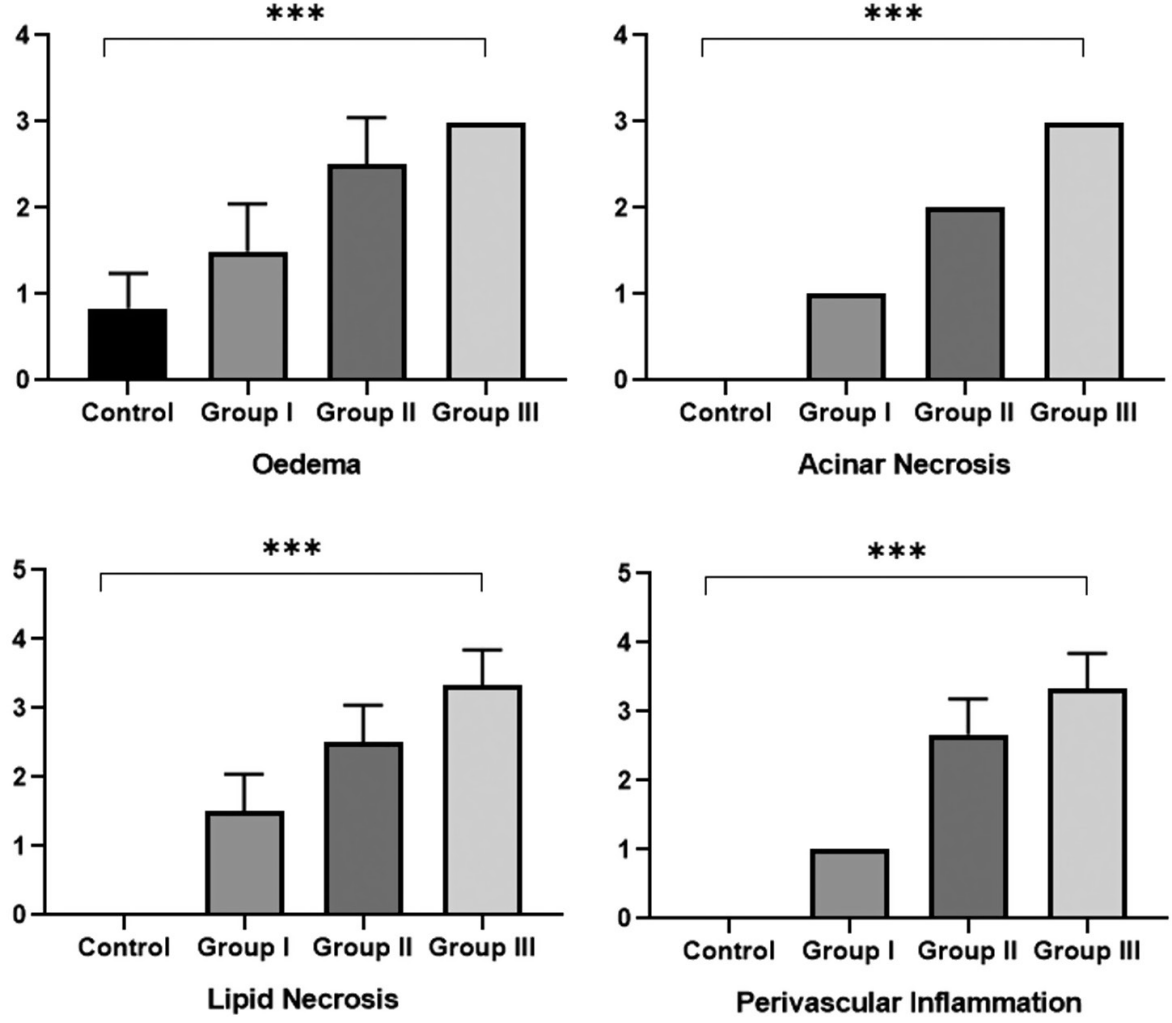
Histopathological findings among study groups.

A total of 24 rats were examined in the study and they were divided into 4 equal groups of 6. The control group was injected 4 times with 1 mL isotonic at 1-hour intervals, 20 μg/kg cerulein in the first group, 30 μg/kg cerulein in the second group, and 40 μg/kg cerulein in the third group. Some laboratory tests and histopathological findings measured and examined in the study groups were taken into comparative analysis, and the findings were also presented in tables and Figures 1 and 2. The ALT values were 44.0 U/L in the control group, 58.5 U/L in the first group, 59.5 U/L in the second group, and 49.0 U/L in the third group. Aspartate transaminase values were 140.0 U/L in the control group, 117.0 U/L in the first group, 130.0 U/L in the second group, and 112.5 U/L in the third group. Amylase values were 410.5 IU/L in the control group, 1946.5 IU/L in the first group, 1170.0 IU/L in the second group, and 1365.0 IU/L in the third group. The lipase values were 11.5 IU/L in the control group, 54.5 IU/L in the first group, 25.5 IU/L in the second group, and 37.0 IU/L in the third group. The HE4 values were 69.8 pmol/L in the control group, 104.7 pmol/L in the first group, 77.5 pmol/L in the second group, and 64.4 pmol/L in the third group (Figure 1).

Table 1 shows the distribution of some laboratory tests among the study groups. While there is no statistically significant difference between the study groups in ALT, AST, and HE4 values, a statistically significant difference was found between amylase and lipase values (*P* < .05). As a result of the post hoc paired comparisons, the significant difference in lipase was observed between the control group and the second group (*P* = .004) and the third group (*P* = .002). The significant difference in amylase was observed between the control group and the first group (*P* = .002), the second group (*P* = .002), and the third group (*P* = .002). The lipase value of the control group was significantly lower than the lipase value of the second and third groups. The amylase value of the control group was significantly lower than all other groups.

In Table 2, the distribution of the histopathological findings between the study groups is presented. The edema scores were calculated as 0.5 in the control group, 1.25 in the first group, 2.5 in the second group, and 3.0 in the third group. Acinar necrosis scores were calculated as 0.0 in the control group, 0.5 in the first group, 2.0 in the second group, and 2.5 in the third group. Fat necrosis scores were calculated as 0.0 in the control group, 1.25 in the first group, 2.25 in the second group, and 3.0 in the third group. Perivascular inflammation scores were calculated as 0.0 in the control group, 1.0 in the first group, 2.5 in the second group, and 3.0 in the third group. Significant differences were found between groups when compared according to acinar necrosis, fat necrosis, and perivascular inflammation scores (Figure 2). It was determined that all scores were found to be different in post hoc paired comparisons (*P* < .05), while the general histopathological findings were the lowest in the control group compared to cerulein at different doses.

## Discussion

Acute pancreatitis is a disease that can present different clinical pictures from mild and self-limiting inflammation to severe infected pancreatic necrosis, multiorgan failure, and high mortality risk. Although gallstones and alcohol are the most common causes in etiology, there are cases of pancreatitis without these 2 reasons. Therefore, systemic approaches are recommended when investigating etiology. Experimental studies rather than clinical studies have come into prominence to understand the mechanisms of acute pancreatitis and to test new pharmacological agents for treatment. Many different experimental acute pancreatitis models have been developed, and the cerulein-induced pancreatitis model, which is one of the models created with secretion stimulants, has been our preference in this study.

Cerulein gives early histopathological findings in rat pancreases similar to human pancreases. Cerulein is used intravenously, intraperitoneally, and subcutaneously.^22^ We used the intraperitoneal injection method in our study. Cerulein causes edematous pancreatitis with edema in the pancreas, vacuolization in acinar cells, leukocyte infiltration, and an increase in serum amylase within a few hours by stimulating the CCK receptors in the pancreatic tissue after its injection. It was observed that as the dose of cerulein increased, damage to the pancreatic parenchyma progressed to necrosis.^23^ In our study, using the Schmidt pancreatic injury scoring system, we showed that the severity of pancreatitis increased with the amount of cerulein given in increasing doses.

As we cannot evaluate histopathological classifications in humans to diagnose pancreatitis and determine the severity of pancreatitis, in addition to physical examination, laboratory findings and radiological imaging methods are used in the diagnosis of acute pancreatitis. The diagnosis of acute pancreatitis was made with the increase in serum amylase or lipase levels (more than three times the normal limits) together with the pain. Imaging methods are necessary when criteria are not met and for monitoring the severity of pancreatitis.^24^

Although amylase and lipase values are elevated in patients with pancreatitis, they are not completely specific to the pancreas. Concentrations in the blood do not correlate with the severity of pancreatitis. Therefore, many scoring methods have been developed in which we try to determine the severity of pancreatitis and the progression of the disease.

The accuracy rate of all these prognostic indicators in pancreatitis prognosis is approximately 70%. Better use of existing predictors (sequence tests, combined tests, or artificial neural network methods) is promising unless there is a new biomarker that ultimately reveals disease severity.^25^ In our study, we used HE4, a biomarker that we can measure its concentration in the blood, to show the severity of pancreatitis. When we looked at the HE4 levels in different pancreatitis severities, we found that the same level of amylase and lipase did not correlate with the severity of pancreatitis and HE4 elevation. We saw that the highest HE4 value was found in the 20 µg/kg cerulein-given group. When we look at our histopathological pancreatitis severity score, we see that the highest HE4 value is in the lowest severity of pancreatitis. This situation made us think that in mild pancreatitis, the HE4 level is high and as the parenchyma damage increases in the pancreas, the serum concentration of HE4 may decrease as the pancreatic necrosis progresses. In our study, we could not look at the HE4 correlation in the progressing period from healthy pancreas to mild pancreatitis, since we did not have another group between the 20 µg/kg cerulein-given group and the control group. We need additional studies to show HE4 levels in experimental pancreatitis with doses given less than 20 µg/kg.

Human Epididymitis Protein 4 is a whey acid protein containing 4 disulfide nuclei. The exact role of HE4 in the human body is unknown. It was first described as a protein secreted from the distal epididymis epithelium in 1991, and it has been suggested to have a function in sperm maturation due to its significant structural similarity to proteinase inhibitors.^26-28^ Many normal tissues, especially the respiratory tract and reproductive epithelium other than the ovary in both sexes, synthesize HE4.^29^

In recent studies, HE4 has been shown to be a potential biomarker for ovarian cancer, lung adenocarcinoma, endometrial cancer, mesothelioma, transitional cell cancers, and breast cancer.^9-15^ In a study, it was stated that HE4 has high sensitivity alone as a marker in the diagnosis of early-stage ovarian cancer, while CA125/HE4 combination has increased sensitivity compared to CA125 alone or HE4 alone.^30^ Human Epididymitis Protein 4 was approved by the US Food and Drug Administration as a serum tumor marker in ovarian carcinomas in 2003.^31^

Although HE4 staining is seen in normal endometrial tissue, HE4 protein expression is increased in endometrial cancer. This increased expression has been associated with the prognosis of endometrial cancer.^32,33^ Moore et al^34^ found increased serum HE4 levels in patients with endometrial cancer in a study in which they compared patients with endometriosis and patients with endometrial cancer. 

The search for new biomarkers in breast cancer still continues. In a study conducted by Gündüz et al^35^ in 2016, they compared breast cancer cases with healthy volunteers and showed that there was a significant level of HE4 elevation in breast cancer patients.

In a study involving 222 serum samples in the urinary tract, HE4 serum levels were found to be significantly higher in patients with transitional cell carcinoma than in the healthy control group and in those with benign urinary disease.^15^

Upper gastrointestinal adenocarcinomas have a particularly poor prognosis. Between 1999 and 2005, only 17% of patients with esophageal cancer, 26% of patients with gastric cancer, and 6% of patients with pancreatic cancer survived 5 years after the disease was identified.^36^ In a gastric study on HE4, they found that HE4 was not secreted in normal gastric mucosa but expressed in mucosa and gastric cancer with intestinal metaplasia.^16^ According to another study related to HE4 and gastric cancer, HE4 expression was found to be associated with disease stage, prognosis, tumor size, and survival.^37^

Pancreatic adenocarcinomas also develop from the formation of noninvasive pancreatic intraepithelial neoplasia (PanIN) lesions. In a 2012 study, HE4 expression was examined in normal, PanIN, and adenocarcinoma samples. Human Epididymitis Protein 4 staining was not observed in normal acinar cells or pancreatic ducts. Strong HE4 staining was observed in pancreatic cancers showing different levels of differentiation. These studies show that the regulation of the extracellular protease inhibitor HE4 is associated with carcinogenesis in the pancreas. They said that although HE4 expression did not have a significant effect on patient outcomes, HE4 could represent an important indicator of the neoplastic process in the stomach and pancreas.^16^

In the study conducted by Huang et al^38^ in 2015, on the expression and diagnostic value of HE4 in pancreatic adenocarcinoma, they presented data showing increased HE4 mRNA and protein expression in pancreatic adenocarcinoma tissues and high HE4 serum levels in patients with pancreatic adenocarcinomas. In their study, it is thought that the height of HE4 may have a higher diagnostic value in the early stage of cancer with biomarker combinations such as CA19-9 and CA15-3, and HE4 is a promising marker for pancreatic adenocarcinomas. Further validation experiments in the larger population are required to determine the efficacy of the ELISA-based HE4 serum assay as a non-invasive tool for the diagnosis and/or screening of pancreatic adenocarcinomas.^38^

## Conclusions

In conclusion, to the best of our knowledge, this is the first experimental study in the literature to have examined the serum HE4 level changes in rats in acute pancreatitis induced with cerulein in a dose-dependent manner. We determined the level of amylase and lipase required to diagnose acute pancreatitis. We found that serum amylase and lipase levels were not correlated with the severity of histopathologically demonstrated pancreatitis. Likewise, we found that the serum HE4 level varied in pancreatitis groups with different severity; the highest HE4 value was in mild pancreatitis but was not correlated with increasing pancreatitis severity. We studied the serum HE4 level in acute pancreatitis, a benign disease, which was studied in search of a biomarker in malignant diseases such as pancreatic and stomach cancer, as there is no example in the literature. Further experimental and clinical studies are needed for new biomarkers that will guide us in the diagnosis and prognosis of acute pancreatitis, which can progress from localized inflammation to systemic disease with a fatal course.

## Figures and Tables

**Table 1.** Laboratory Findings of Study Groups

Parameters	Control (1 mL Isotonic) (n = 6)	Group 1 (20 µm/kg Cerulein) (n = 6)	Group 2 (30 µm/kg Cerulein) (n = 6)	Group 3 (40 µm/kg Xerulein) (n = 6)	*P*
ALT (U/L)	44.0 (40-54)	58.5 (37-78)	59.5 (45-108)	49 (42-61)	.094
AST (U/L)	140 (87-166)	117 (76-133)	130 (107-188)	112.5 (71-134)	.134
Lipase * (IU/L)	11.5 (7-17)	54.5 (9-102.0)	25.5 (16.0-41.0)	37 (19.0-109.0)	. **010**
Amylase ** (IU/L)	410.5 (339-615)	1946.5 (631-3908)	1170 (1035.0-1745.0)	1365 (928.0-3034.0)	. **003**
HE4 (pmol/L)	69.8 (59.2-87.6)	104.7 (57.9-131.5)	77.5 (58.4-117.2)	64.4 (52.7-77.0)	.071

The results were presented as median (min-max). The significant difference in amylase was observed between the control group and the 1st group. ALT, alanine aminotransferase; AST, aspartate aminotransferase; HE4, Human Epididymitis Protein-4.

*Control < Groups 1-3, **Control < Groups 1-3.

**Table 2.** Histopathological Findings of the Study Groups

Parameters	Control (1 mL Isotonic) (n = 6)	Group 1 (20 µm/kg Cerulein) (n = 6)	Group 2 (30 µm/kg Cerulein) (n = 6)	Group 3 (40 µm/kg Cerulein) (n = 6)	*P*
Edema	0.5 (0.0-0.5)	1.25 (1.0-1.5)	2.5 (2.0-2.5)	3.0 (3.0-3.5)	**<.001**
Acinar necrosis	0.0 (0.0-0.0)	0.5 (0.5-0.5)	2.0 (1.5-2.0)	2.5 (2.0-3.0)	**<.001**
Lipid necrosis	0.0 (0.0-0.0)	1.25 (0.5-1.5)	2.25 (1.5-2.5)	3.0 (2.5-3.5)	**<.001**
Perivascular inflammation	0.0 (0.0-0.0)	1.0 (1.0-1.0)	2.5 (2.0-2.5)	3.0 (3.0-3.5)	**<.001**

The results were presented as median (min-max).

Significant differences were found between control and the cerulein groups when compared according to edema, acinar necrosis, lipid necrosis and perivascular inflammation scores.
